# Neoadjuvant chemotherapy before radical prostatectomy for locally advanced prostate cancer: Protocol for a systematic review and meta-analysis

**DOI:** 10.1097/MD.0000000000017060

**Published:** 2019-08-30

**Authors:** Tianhai Lin, Xiong Yang, Lina Gong, Hang Xu, Shi Qiu, Ruichao Yu, Sheng Sun, Liangren Liu, Peng Zhang, Ping Han, Jingqiu Cheng, Lu Yang, Qiang Wei

**Affiliations:** a Department of Urology and National Clinical Research Center for Geriatrics; b Key Laboratory of Transplant Engineering and Immunology, West China Hospital, Sichuan University, Chengdu, Sichuan Province, China; c Department of Pulmonary, Brigham and Women's Hospital; d Massachusetts General Hospital Cancer Center, Harvard Medical School, Boston, MA.

**Keywords:** chemotherapy, docetaxel, neoadjuvant, prostate cancer, taxanes

## Abstract

**Background:**

To evaluate the effectiveness and safety of neoadjuvant chemotherapy (NAC) for locally advance prostate patients undergoing radical prostatectomy.

**Methods:**

PubMed/Medline, EMBASE, Web of Science, Ovid, Web of Knowledge, and Cochrane Library will be searched for studies related to the topic. The identification, inclusion and exclusion flow charts will be conducted according to PRISMA guidelines. The identified reports will be critically appraised using GRADE approach. Bias and heterogeneity of included studies will be assessed, and outcome measurements from individual studies will be combined with 95% confidence interval using a fixed- or random-effects model if qualified.

**Results:**

This study will provide evidence and data on the tolerance and efficacy of NAC followed by radical prostatectomy (RP).

**Conclusion:**

The application of taxanes-based chemotherapy has been widened to metastatic hormone sensitive prostate cancer in recent years. To be more vigorous, whether neoadjuvant administration of these cytotoxic agents can improve the outcome of RP in locally advance prostate cancer patients has been explored. This study aims to synthesis data regarding the adverse effect, response rate, recurrence, and survival from multiple trials, and to guide the healthcare practitioners using an evidence-based approach.

## 1 Introduction

Prostate cancer has the second-high prevalence among cancer in men, which has become a severe medical problem.^[1]^ Although patients diagnosed with localized prostate cancer may survival for a long time without progression, high-risk disease defined using D’Amico criteria (prostate-specific antigen [PSA] ≥ 20 ng/dL, Gleason score higher than 7, and clinical state ≥ T2c) are prone to recur and metastasize after local therapy.^[2]^ Radical prostatectomy (RP) only is far from adequate for the locally advanced prostate cancer, including disease with pelvic lymph node involvement. Instead, a multimodal approach is implemented, with neoadjuvant or adjuvant therapies such as pelvic radiation therapy, androgen deprivation therapy, and chemotherapy, to minimalize the risk of positive surgical margin and recurrence.

Neoadjuvant androgen deprivation therapy (ADT) before RP has been extensively tested and shows no significant improvement of long-term outcome for locally advanced prostate cancer.^[3]^ Several studies have admitted that the decreasing in serum PSA and prostate volume was observed, and the optimal duration of neoadjuvant ADT may be at least 8 months for clinical benefit.^[4,5]^ However, the extended waiting is likely to arise anxious in patients due for RP, or even worse result in disease progression for androgen-independent prostate cancer.

Chemotherapy has been the standard of care for castration-resistant prostate cancer (CRPC) since the development of the taxanes.^[6,7]^ In recent years, its application has been widened to metastatic hormone sensitive prostate cancer, on the basis of pivotal randomized phase III trials in this area: GETUG15, CHAARTED, and STAMPEDE.^[8]^ The question remains whether neoadjuvant administration of cytotoxic agents with RP can improve perioperative outcome and long-term survival. Many of the studies have small sample size or are single-arm trials, which makes them less convincing. Therefore, the present study aims to synthesize current available evidences on the tolerance and efficacy of NAC followed by RP, and to combine data from multiple studies using meta-analysis.

## 2 Methods

This protocol is conducted according to Preferred Reporting Items for Systematic Reviews and Meta-Analyses Protocols (PRISMA-P) guidelines and registered on the international prospective register of systematic reviews (PROSPERO registration number: CRD42019123375. Available at: http://www.crd.york.ac.uk/prospero/). We will document essential protocol amendments in the full review and update information in the registry. This study has been approved by the Ethics Committee of West China Hospital, Sichuan University (Chengdu, China).

### 2.1 Evidence acquisition

Authenticated databases including PubMed/Medline, Embase, Web of Science, Ovid,Web of Knowledge, and Cochrane Library will be extensively searched for articles written in English and published from January 2000 to December 2018. MeSH words and free words with the following searching strategy: “prostate cancer” AND “neoadjuvant” AND (“docetaxel” OR “taxanes” OR “chemotherapy”) will be used in the literature search.

Two reviewers will screen search results independently for duplicates and irrelevant articles, which will be removed for further analysis. Remain records will be interrogated to acquire full text or raw data, and case reports, meeting proceedings, editorials, reviews, or letters will be excluded.

The inclusion criteria for studies are: randomized controlled trials or single-arm trials, using chemotherapy combined with or without ADT neoadjuvantly, including 10 or more participants, and having reported adverse effect, perioperative outcome, recurrence or survival. Local therapy other than RP is not allowed and the study will be excluded.

The consensus on the evidence acquisition will be reported, and a third reviewer will be consulted if required.

### 2.2 Data extraction

We will extract following information from qualified studies: title, author, nationality, department, ethnicity, study design, age of the patients (both the experimental and control group), enrollment year, regimens of chemotherapy, administration of combination regimen, and parameters of correlated outcomes including adverse effect, response rate, and recurrence.

Two reviewers will generate an electric data table containing extracted parameters. Discrepancies will be resolved by a third reviewer if necessary.

### 2.3 Quality evaluation

The quality of selected studies will be appraised using GRADE approach, which evaluates factors including study design, consistency of the results, use of resources, etc.^[9]^

### 2.4 Publication bias

If more than 10 studies include data qualified for synthesis, we will use “funel plot” to detect the risk of publication bias. Otherwise, Begg test and Egger test will be implemented. These tests will be performed using STATA 14.2 (StataCorp).

### 2.5 Heterogeneity assessment

The I^2^ statistics and Galbraith plot will be used to determine the heterogeneity of included studies. When I^2^ < 50%, a fixed-effects model will be used in following meta-analysis, otherwise a random-effects model will be applied. If the heterogeneity is high, the Galbraith plot will identify the outliers and a sensitivity analysis will be performed. With adequate data, we will perform subgroup analyses based on different patients, regimens and controls to abrogate the impact of heterogeneity.

### 2.6 Statistical analysis

Relative risk will be used to analyze dichotomous outcomes including adverse effect, response rates, and recurrence. Survival outcomes will be derived and reported as hazard ratios. Outcome measurements from individual studies will be presented with 95% confidence interval and combined through meta-analysis using STATA 14.2 (StataCorp).

## 3 Discussion

This systematic review will assess the safety and the effectiveness of NAC for locally advanced prostate cancer. In addition to ADT, chemotherapy has not only shown promising effect on CRPC patients, but also improved the outcome of patients with androgen-sensitive prostate cancer. Although neoadjuvant ADT has significant impacts on surgical outcomes of locally advanced disease, such as decreasing PSA and prostate volume, lower rates of positive surgical margin and lymph node positivity, and down-staging pathological T stage, there is no clear improvement in the biochemical recurrence and survival.^[10]^ As ADT can only suppress cancer cells depending on androgen and may induce the epithelial–mesenchymal transition, it is possible a portion of androgen-independent cancer cells have been mobilized during the perioperative period and invisible to PSA and imaging detection, which leads to recurrence and metastasis. In that regards, NAC have been considered to optimize the management of high-risk and locally prostate cancer. Although conflicts still exist on the survival benefit of NAC, as well as the dosage of taxanes and the combination regimens, NAC will improve the outcome of a subset of patients with locally advanced prostate cancer.

## Author contributions

Conceptualization: Tianhai Lin, Lu Yang, Qiang Wei.

Data curation: Tianhai Lin, Lina Gong.

Formal analysis: Tianhai Lin, Xiong Yang.

Funding acquisition: Jingqiu Cheng, Qiang Wei.

Investigation: Xiong Yang, Lina Gong.

Methodology: Lu Yang, Liangren Liu, Hang Xu

Project administration: Peng Zhang, Ping Han.

Software: Tianhai Lin, Sheng Sun, Shi Qiu

Supervision: Jingqiu Cheng, Qiang Wei.

Writing – original draft: Tianhai Lin, Xiong Yang.

Writing – review & editing: Tianhai Lin, Ruichao Yu.

Tianhai Lin, Lu Yang and Qiang Wei will guarantee the finish of this review.

**Conceptualization:** Qiang Wei, Tianhai Lin.

**Data curation:** Lina Gong.

**Formal analysis:** Tianhai Lin, Xiong Yang.

**Funding acquisition:** Qiang Wei.

**Investigation:** Xiong Yang, Lina Gong.

**Methodology:** Hang Xu, Liangren Liu, Lu Yang.

**Project administration:** Peng Zhang, Ping Han.

**Software:** Tianhai Lin, Shi Qiu, Sheng Sun.

**Supervision:** Qiang Wei, Jingqiu Cheng.

**Writing – original draft:** Tianhai Lin, Xiong Yang.

**Writing – review & editing:** Tianhai Lin, Ruichao Yu.
